# Association between insulin resistance and uncontrolled hypertension and arterial stiffness among US adults: a population-based study

**DOI:** 10.1186/s12933-023-02038-5

**Published:** 2023-11-09

**Authors:** Liao Tan, Yubo Liu, Jie Liu, Guogang Zhang, Zhaoya Liu, Ruizheng Shi

**Affiliations:** 1 Department of Cardiology, Third Xiangya Hospital, Central South University, Hunan, China; 2 Department of Cardiovascular Medicine, Xiangya Hospital, Central South University, Changsha, Hunan China; 3 Department of the Geriatrics, Third Xiangya Hospital, Central South University, Hunan, China

**Keywords:** Triglyceride-glucose index, Insulin resistance, Hypertension, NHANES, Arterial stiffness

## Abstract

**Background:**

Prior research has established the correlation between insulin resistance (IR) and hypertension. While the association between triglyceride-glucose (TyG) index, a reliable surrogate marker of IR, and uncontrolled hypertension as well as arterial stiffness among individuals with hypertension remains undisclosed.

**Methods:**

In this study, a total of 8513 adults diagnosed with hypertension from the National Health and Nutrition Examination Survey 1999–2018 were included. The primary outcome of the study are arterial stiffness (represented with estimated pulse wave velocity, ePWV) and uncontrolled hypertension. Logistic regression model, subgroup analysis, restricted cubic spine, and smooth curve fitting curve were conducted to evaluate the association between the IR indicators and uncontrolled hypertension and arterial stiffness in individuals with hypertension.

**Results:**

Among included participants, the overall prevalence of uncontrolled hypertension was 54.3%. After adjusting for all potential covariates, compared with the first quartile of TyG index, the risk of uncontrolled hypertension increased about 28% and 49% for participants in the third quartile (OR, 1.28; 95% CI 1.06–1.52) and the fourth quartile (OR, 1.49; 95% CI 1.21–1.89) of TyG index, respectively. The higher OR of TyG index was observed in participants taking antihypertensive medication [fourth quartile versus first quartile (OR, 2.03; 95% CI 1.37–3.11)]. Meanwhile, we explored the potential association between TyG index and arterial stiffness and found that TyG index was significantly associated with increased arterial stiffness (β for ePWV, 0.04; 95% CI 0.00–0.08; P = 0.039). However, traditional IR indicator HOMA-IR showed no significant positive correlation to uncontrolled hypertension as well as arterial stiffness in US adults with hypertension.

**Conclusion:**

Elevated levels of the TyG index were positive associated with prevalence of uncontrolled hypertension and arterial stiffness among US adults with hypertension.

**Supplementary Information:**

The online version contains supplementary material available at 10.1186/s12933-023-02038-5.

## Introduction

Hypertension is a major risk factor for cardiovascular diseases (CVD) and has emerged as a primary cause of mortality and disability-adjusted life years worldwide [1, 2]. As the increasing hypertension awareness, therapy methods of hypertension have been improved over time, but it remains suboptimal in blood pressure control and vascular function preservation [3]. In the United States, the prevalence of controlled blood pressure declined from 53.8% in 2013–2014 to 43.7% in 2017–2018 [3]. There is strong observational evidence that uncontrolled hypertension is associated with a large global burden of CVD [4]. Specifically, every 20 mmHg elevation in systolic blood pressure and 10 mmHg elevation in diastolic blood pressure significantly increased the risk of CVD mortality by about twofold [5, 6]. Unsatisfactory blood pressure control implies that there is a significant potential to mitigate CVD events through improved blood pressure management [7].

A poorer blood pressure control means a greater BP variability, that is associated with vascular remodelling and subsequent arterial stiffness [8, 9]. Meanwhile, arterial stiffening among hypertensive patients is also responsible for the poorer response to antihypertensive therapy and with greater hypertension-mediated organ damage [10, 11]. Consequently, identifying and evaluating individuals at higher risk of uncontrolled blood pressure and higher arterial stiffness is imperative, facilitating disease prevention and slowing the progression of CVD [12, 13]. The carotid-femoral pulse wave velocity (cfPWV) is the most well-studied marker used to represent arterial stiffness [14]. The quantification of cfPWV necessitates the utilization of specialized instrumentation, which has yet to achieve widespread integration into clinical practice. Thus, the streamlining of this technology and exploration of novel, cost-effective methodologies for assessing or approximating arterial stiffness is poised to expedite its assimilation within the clinical domain. In pursuit of this objective, multiple initiatives have been undertaken to evaluate arterial stiffness. These methods comprise the derivation of mathematical equations incorporating age and mean blood pressure (MBP) as variables, as well as the application of artificial intelligence techniques [15]. These estimations of cfPWV have manifested robust correlations with in vivo assessments, while estimated PWV (ePWV) has exhibited an independent predictive capacity in contrast to conventional risk scoring systems [16]. Consequently, ePWV were setted as a main outcome represent arterial stiffness in this study.

Accumulating evidence suggested that insulin resistance (IR), the inability of insulin to increase cellular glucose uptake and utilization, is an important contributing factor to cardiovascular diseases, including hypertension and coronary artery disease [17–20]. Patients with diabetes frequently present with concomitant hypertension, and those with hypertension at the time of diabetes diagnosis exhibit a higher risk of all-cause and CVD mortality than diabetic patients with normotension [21, 22]. Meanwhile, fasting and postprandial insulin levels often higher in the untreated essential hypertensive patients than in normotensive individuals [23]. To this point, IR may play an important role in the increasing propensity for development of essential hypertension. In addition, as IR contributes to the dysfunction of the sympathetic nervous system and the renin–angiotensin–aldosterone system, an integrated pharmacotherapeutic strategy should be applied to reduce cardiovascular risk in hypertensive patients with IR [24].

The triglyceride-glucose (TyG) index, a parameter calculated from the fasting blood glucose and triglyceride levels, has been identified as a representative and reliable biomarker of IR. Previous studies suggested that a high TyG index is associated with high risk of cardiovascular events [25–29]. Additionally, elevated TyG index has been found to be positively associated with a higher prevalence of hypertension and demonstrates superior discriminative ability for hypertension compared with some traditional parameters such as lipid and glycemic parameters [19]. Consequently, the TyG index might serve as potentially valuable marker in hypertension. However, it is unclear whether the TyG index show a better performance in predicting uncontrolled hypertension and arterial stiffness than traditional IR in U.S. patients with hypertension.

The National Health and Nutrition Examination Survey (NHANES) is designed to provide nationally representative estimates of health and disease in the non-institutionalized population. Hence, using the data from the NHANES 1999–2018, our study aims to explore the association of the TyG index and other IR indicators with uncontrolled hypertension and arterial stiffness in US adults with hypertension.

## Methods

### Study design and population

**Fig. 1:**
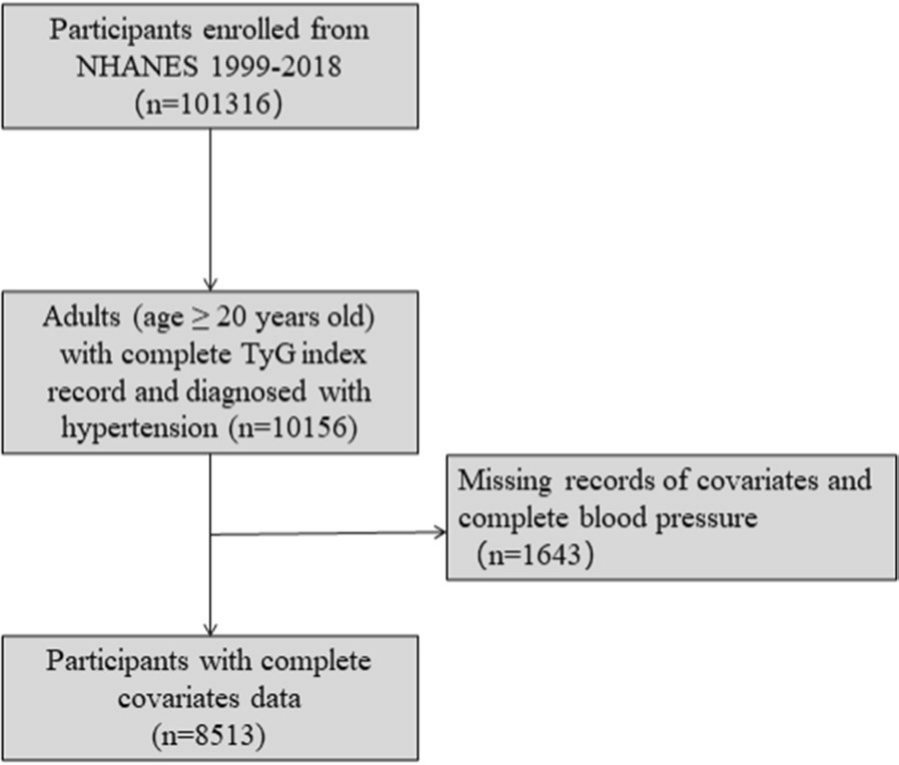
Flowchart of study population

Data of this cross-sectional study were collected from the NHANES (10 cycles from 1999 to 2018), a periodic and representative program conducted by the National Center for Health Statistics to collect data about the nutrition and health status from the US. With the sampling design of the program, estimates are representative of the US population. The program has been approved by the National Center for Health Statistics Ethics Review Board. Written informed consent was obtained from each participant in the program, so specific written consent was not required for further analysis. A total of 8513 participants diagnosed with hypertension in NHANES 1999–2018 were enrolled in the final analysis. The details of the inclusion and exclusion process are showed in Fig. 1.

### Data collection and definitions

Data on demographic (age, sex, ethnicity, education level, family income to poverty ratio), health-related behavior (physical activity, smoking status, and alcohol consumption), health condition (hypertension, diabetes, hyperlipidemia, cardiovascular disease, chronic kidney disease, and medications), physical measurements as well as laboratory data were collected and administered by trained staffs according to standardized questionnaires and mobile examination centers. Blood pressure was measured by trained medical professionals using a mercury sphygmomanometer with an appropriately sized cuff. Three consecutive results of measurements were obtained after participants rest quietly for 5 min. Measurements were made in a sitting position and using the right arm unless there were special circumstance. Average blood pressure of three measurements were calculated and used for further analysis. Hypertension was defined as systolic blood pressure of 140 mmHg or higher, diastolic blood pressure level of 90 mmHg or higher, or participants self-reported use of antihypertensive medications or a doctor’s diagnosis of hypertension.

BMI was calculated as weight in kilograms divided by height in meters squared. Physical activity (PA) data was collected using weekly physical activity participation information collected by the Global Physical Activity Questionnaire (GPAQ) created from the World Health Organization. PA data were analyzed following the World Health Organization analysis guide and were presented with metabolic equivalent (MET) minutes of moderate to vigorous physical activity per week. MET score for specific activities were calculated based on the activity type and intensity and the NHANES offers the recommended MET values for each kind of activity. PA was based on the MET values of type, frequency, and duration of activities per week, which was calculated using the following formula: PA (MET-min/week) = MET × weekly frequency × duration of each PA [30]. PA = 0 denotes participants who do not engage in any PA, else, it means that participants have constant or intermittent PA. The following conditions would be defined as diabetes: (a) HbA1c > 6.5% or fasting plasma glucose level ≥ 7.0 mmol/L or 2 h plasma glucose ≥ 11.1 mmol/L or random blood glucose ≥ 11.1 mol/L; (b) self-reported use of antidiabetic medications or a doctor’s diagnosis diabetes. Low-density lipoprotein (LDL) was calculated by total cholesterol minus high-density lipoprotein -cholesterol minus triglycerides/5 in mg/dl. The following conditions would be defined as having hyperlipidemia: (a) total cholesterol ≥ 5.18 mmol/L or LDL ≥ 3.37 mmol/L; (b) if high-density lipoprotein (HDL) < 1.04 mmol/L (male) or < 1.30 mmol/L (female); (c) they self-reported use of cholesterol-lowering medications or a doctor’s diagnosis hyperlipidemia. Estimation of glomerular filtration rate (eGFR) was calculated using the Chronic Kidney Disease Epidemiology Collaboration (CKD-EPI) creatinine equation. Albuminuria was defined as the ratio of urine albumin to creatinine being 30 mg/g or above. Participants were classified as having chronic kidney disease if eGFR < 90 mL/min/1.73m^2^ or the presence of albuminuria. Participants who self-reported a history of coronary heart disease, angina, or myocardial infarction were classified as having coronary heart disease from the Questionnaire.

Data on deaths were obtained by linking the NHANES database with the National Death Index. All-cause mortality was defined as any cause for death and CVD mortality was classified by the International Statistical Classification of Disease, 10th Revision codes I00 to I09, I11, I13, I20 to I51, and I60 to I69.

### Exposure variable and study outcomes

In this study, the primary exposure variable was the IR, which was represented by the TyG index and HOMA-IR. The TyG index was calculated using the formula ln $[{\text{fasting triglyceride}}\left( {{\text{mg}}/{\text{dL}}} \right) \, \times {\text{ fasting glucose}}\left( {{\text{mg}}/{\text{dL}}} \right)/{2}]$ [20]. The Homeostatic Model Assessment of IR (HOMA-IR) was conducted in accordance with fasting serum glucose and insulin levels as follows: $\left[ {{\text{fasting insulin }}\left( {{\mu U}/{\text{mL}}} \right) \, \times {\text{ fasting glucose }}\left( {{\text{mmol}}/{\text{L}}} \right)} \right]/{22}.{5}$ [31]. In addition, The Homeostatic Model Assessment of insulin sensitivity (HOMA-IS) were calculated according to the standard formula: ${22}.{5}/ \, [{\text{fasting glucose }}\left( {{\text{mmol}}/{\text{L}}} \right)\, \times \,{\text{fasting insulin }}\left( {{\text{mIU}}/{\text{L}}} \right)]$ [32].

The primary outcome assessed was uncontrolled hypertension and arterial stiffness. Uncontrolled hypertension was defined as systolic blood pressure higher than 140 mmHg and/or diastolic blood pressure higher than 90 mmHg among included individuals with hypertension [3, 33]. Arterial stiffness was represented with ePWV. The equation derived from the Reference Values for Arterial Stiffness Collaboration was used to calculate ePWV. According to the equation, ePWV was calculated from age and mean blood pressure (MBP): [16, 34].

$$\begin{gathered} {9}.{587 } - \, 0.{4}0{2 } \times {\text{ age }} + { 4}.{56}0 \, \times { 1}0^{ - {3}} \times {\text{ age}}^{2} - { 2}.{621 } \times { 1}0^{ - {5}} \times {\text{ age}}^{2} \times {\text{ MBP }} + { 3}.{176 } \times { 1}0^{ - {3}} \hfill \\ \times \;{\text{age }} \times {\text{ MBP }} - { 1}.{832 } \times { 1}0^{ - {2}} \times {\text{ MBP}}.{\text{ MBP was calculated as diastolic blood pressure }} + \, \hfill \\ 0.{4 } \times \, \left( {{\text{systolic blood pressure }} - {\text{ diastolic blood pressure}}} \right) \hfill \\ \end{gathered}$$

### Statistical analysis

All analyses were calculated using appropriate sample weights to obtain accurate estimates representing the civilian non-institutionalized US population. Mean and standard error (mean ± SE) were calculated for continuous variables, while categorical variables were presented in counts (weighted proportion). TyG index have no recommended cut-off value to diagnose IR. Hence all included participants were divided into four equal groups by the TyG index followed previous research [35, 36]. This cut-off values were followed in further statistical analysis. Student’s t test, the Mann–Whitney U test, and chi-square tests were performed to analyze the differences between different groups. Multivariable logistic regression models were used to evaluate odds ratios (OR) and 95% confidential intervals (CI) for the association between different quartiles of TyG index and blood pressure uncontrolled prevalence. The first quartile was used as the reference group. Two models were conducted for the analysis. Model 1 adjusted for age (continuous), sex (male or female), race (Mexican American, non-Hispanic Black, non-Hispanic White, Other Hispanic, or Other Race), education (less than high school, high school, or more than high school). Mode 2 further adjusted for BMI (continuous), smoking status (never, former, or current), alcohol consumption (never, former, mild, moderate, or heavy), diabetes (no or yes), hyperlipidemia (no or yes), cardiovascular disease (no or yes), chronic kidney disease (no or yes), anti-hypertensive agents (no or yes), anti-diabetic agents (no or yes), anti-hyperlipidemia agents (no or yes). A test for linear trend was performed with the use of quintiles of the TyG index values as a continuous variable by assigning the median values of the quintiles to the TyG index. Moreover, restricted cubic spline (RCS) analysis was performed to detect the shape of dose–response relationships of TyG index with uncontrolled hypertension. We used the R rms package anova function to estimate P value for nonlinearity. If P value for non-linearity were < 0.05, that indicated dose–response relationship in a non-linear manner. Finally, the interaction of the TyG with different subgroups of uncontrolled hypertension was assessed by including stratification analysis and interaction tests in the regression model. Moreover, the effect dose response between the TyG index and ePWV was evaluated by a generalized additive model and fitting curve (penalized spline method). All analyses were performed with R software (http://www.R-project.org). P < 0.05 was considered statistically significant.

## Results

### Participants characteristics

In the present study, a total of 8513 participants diagnosed with hypertension (4223 females and 4290 males) were enrolled in the analysis. The baseline characteristics of the study participants according to TyG index quartiles (quartile 1: n = 2128, 6.76 ≤ TyG index ≤ 8.37; quartile 2: n = 2128, 8.38 ≤ TyG index ≤ 8.79; quartile 3: n = 2128, 8.80 ≤ TyG index ≤ 9.21; quartile 4: 9.22 ≤ TyG index ≤ 12.55) are shown in Table 1. Compared with the lowest quartile of TyG index, participants with higher quartile of TyG index were more likely to be older, male, non-Hispanic White, less educated, smoker, over-weighted, with insulin resistance, and tended to have higher blood pressure, higher prevalence rates of diabetes, hyperlipidemia, cardiovascular disease, and chronic kidney disease. Moreover, participants in the highest quartile of TyG index had an increased ePWV and higher percentage of all-cause and CVD mortality rate compared with lower quartile of TyG index in the follow-up duration (Table 1).

**Table 1:** Baseline Characteristics of 8513 Participants According to TyG index in the NHANES 1999–2018

Characteristics	No. (weighted, %)				Total	P-value
	Quartiles of TyG index					
	Q1 (n = 2128) (6.76–8.37)	Q2 (n = 2128) (8.38–8.79)	Q3 (n = 2128) (8.80–9.21)	Q4 (n = 2129) (9.22–12.55)		
TyG index	8.04 ± 0.01	8.59 ± 0.00	8.99 ± 0.00	9.70 ± 0.01	8.84 ± 0.01	< 0.001
Age, years	55.02 ± 0.48	57.55 ± 0.49	58.15 ± 0.39	56.83 ± 0.38	56.91 ± 0.24	< 0.001
Sex						< 0.001
Female	1097 (53.42)	1073 (51.06)	1100 (51.37)	953 (42.68)	4223	
Male	1031 (46.58)	1055 (48.94)	1028 (48.63)	1176 (57.32)	4290	
Ethnicity						< 0.001
Non-Hispanic White	853 (65.11)	1046 (73.65)	1095 (75.28)	1095 (76.64)	4089	
Non-Hispanic Black	846 (22.00)	498 (11.15)	351 (8.47)	281 (6.57)	1976	
Mexican American	180 (4.00)	280 (5.27)	338 (5.47)	437 (6.50)	1235	
Other Race	132 (5.52)	160 (6.36)	152 (5.45)	136 (5.93)	580	
Other Hispanic	117 (3.37)	144 (3.57)	192 (5.33)	180 (4.35)	633	
Education level						< 0.001
Less than high school	211 (5.48)	288 (6.97)	309 (7.31)	395 (8.92)	1203	
High school	836 (35.65)	858 (39.04)	857 (39.61)	901 (41.07)	3452	
More than high school	1081 (58.87)	982 (533.99)	962 (53.08)	833 (50.01)	3858	
Alcohol consumption						0.03
Never	316 (11.50)	319 (11.66)	340 (13.17)	348 (13.28)	1323	
Former	438 (17.10)	465 (17.70)	492 (19.92)	559 (22.48)	1954	
Mild	764 (38.50)	798 (40.67)	742 (39.12)	674 (36.57)	2978	
Moderate	304 (17.39)	256 (14.60)	230 (12.09)	214 (12.58)	1004	
Heavy	306 (15.50)	290 (15.37)	324 (15.69)	334 (15.09)	1254	
Smoking status						< 0.001
Never	1135 (52.51)	1052 (49.37)	1089 (49.62)	945 (43.53)	4221	
Former	605 (28.22)	692 (33.47)	686 (32.58)	750 (35.71)	2733	
Current	388 (19.26)	384 (17.16)	353 (17.80)	434 (20.76)	1559	
PIR	3.06 ± 0.06	3.04 ± 0.06	2.99 ± 0.06	2.99 ± 0.06	3.02 ± 0.04	0.72
MET, min/week	3454.14 ± 197.46	3126.10 ± 187.70	2478.83 ± 177.88	2833.40 ± 156.55	2978.21 ± 106.16	< 0.001
BMI, kg/m^2^	28.59 ± 0.20	30.19 ± 0.18	31.50 ± 0.23	32.47 ± 0.20	30.71 ± 0.11	< 0.001
SBP, mm/Hg	131.91 ± 0.50	132.76 ± 0.53	133.15 ± 0.62	134.24 ± 0.46	133.03 ± 0.30	0.003
DBP, mm/Hg	72.33 ± 0.45	73.19 ± 0.45	73.25 ± 0.41	74.37 ± 0.40	73.30 ± 0.25	0.003
Uncontrolled hypertension	933 (38.75)	968 (40.45)	986 (41.85)	1003 (43.10)	3890	< 0.001
Blood glucose	98.53 ± 0.38	103.98 ± 0.45	111.49 ± 0.71	138.65 ± 1.74	113.33 ± 0.59	< 0.001
Insulin, uu/mL	10.33 ± 0.44	12.79 ± 0.25	16.05 ± 0.36	22.14 ± 0.61	15.38 ± 0.25	< 0.001
HbA1c, %	5.48 ± 0.01	5.61 ± 0.01	5.80 ± 0.03	6.44 ± 0.05	5.83 ± 0.02	< 0.001
Triglyceride, mg/dL	65.77 ± 0.54	105.90 ± 0.48	149.39 ± 0.85	275.37 ± 4.54	150.04 ± 1.84	< 0.001
Cholesterol, mg/dL	183.98 ± 1.15	196.55 ± 1.16	201.36 ± 1.14	210.93 ± 1.53	210.93 ± 1.53	< 0.001
HOMA-IR	2.58 ± 0.11	3.35 ± 0.07	4.53 ± 0.14	8.03 ± 0.28	4.65 ± 0.09	< 0.001
HOMA-IS	0.71 ± 0.02	0.49 ± 0.01	0.38 ± 0.01	0.26 ± 0.01	0.45 ± 0.01	< 0.001
ePWV (m/s)	9.20 ± 0.06	9.54 ± 0.07	9.56 ± 0.06	9.43 ± 0.05	9.47 ± 0.03	< 0.001
Anti-hypertensive agents						< 0.001
No	1708 (82.03)	1652 (77.63)	1595 (75.43)	1585 (74.85)	6540	
Yes	420 (17.97)	476 (22.37)	533 (24.57)	544 (25.15)	1973	
Anti-diabetic agents						< 0.001
No	1946 (93.82)	1873 (91.43)	1730 (85.04)	1375 (70.04)	6924	
Yes	182 (6.18)	255 (8.57)	398 (14.96)	754 (29.96)	1589	
Anti-hyperlipidemia agents						< 0.001
No	1572 (75.00)	1484 (69.85)	1399 (65.92)	1323 (61.96)	5778	
Yes	556 (25.00)	644 (30.15)	729 (34.08)	806 (38.04)	2735	
Diabetes						< 0.001
No	1805 (88.60)	1669 (83.12)	1434 (72.64)	927 (52.59)	5835	
Yes	323 (11.40)	459 (16.88)	694 (27.36)	1202 (47.41)	2678	
Hyperlipidemia						< 0.001
No	797 (38.39)	444 (20.01)	182 (8.05)	40 (1.26)	1463	
Yes	1331 (61.61)	1684 (79.99)	1946 (91.95)	2089 (98.74)	7050	
CVD						< 0.001
No	1761 (85.34)	1726 (83.83)	1693 (82.67)	1618 (78.38)	6798	
Yes	367 (14.66)	402 (16.17)	435 (17.33)	511 (21.62)	1715	
CKD						< 0.001
No	1577 (79.48)	1546 (77.48)	1473 (74.97)	1302 (69.19)	5898	
Yes	551 (20.52)	582 (22.52)	655 (25.03)	827 (30.81)	2615	
All-cause mortality						< 0.001
Alive	1679 (83.23)	1554 (78.96)	1552 (78.71)	1488 (76.27)	6273	
Death	449 (16.77)	574 (21.04)	576 (21.29)	641 (23.73)	2240	
CVD mortality						0.01
Alive	1985 (94.91)	1929 (92.79)	1924 (93.08)	1905 (91.82)	7743	
Death	143 (5.09)	199 (7.21)	204 (6.92)	224 (8.18)	770	

All values were presented as mean ± SE, or counts (weighted, proportion)

TyG index, triglyceride-glucose index; NHANES, National Health and Nutrition Examination Survey; Q, quartiles; BMI, body mass index; MET, metabolic equivalent; PIR, family income to poverty ratio; SBP, systolic blood pressure; DBP, diastolic blood pressure; HbA1c, hemoglobin type A1C; ePWV, estimated pulse wave velocity; CVD, cardiovascular disease; CKD, chronic kidney disease

### Association between IR and uncontrolled hypertension among hypertension adults

**Fig. 2:**
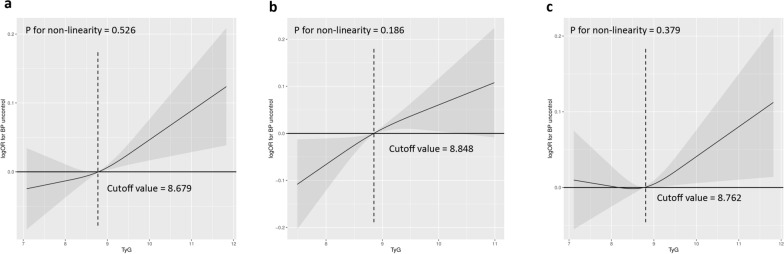
The restricted cubic spline of TyG index and the incidence of uncontrolled hypertension. Plane (**a**): total hypertensive participants; Plane (**b**): hypertensive participants with anti-hypertensive medication; Plane (**c**): hypertensive participants without anti-hypertensive medication. The horizontal solid line represents the logOR = 0. The black curve shows the value of logOR. The grey shaded area represents the 95 CI. Adjusted for age, sex, ethnicity, education, BMI, smoking status, alcohol consumption, diabetes, hyperlipidemia, cardiovascular disease, chronic kidney disease, anti-hypertensive agents, anti-diabetic agents, and anti-hyperlipidemia agents

Among all included hypertension patients, there was a significant positive association of the TyG index with uncontrolled hypertension. After adjusting for covariates, compared with the reference group (quartile 1), the risk of uncontrolled hypertension was increased by about 28% and 49% for participants in the third quartile (OR 1.28, 95% CI 1.06–1.52) and the fourth quartile (95% CI 1.21–1.89) of TyG index, respectively (Table 2). Figure 2a shows restricted cubic splines indicating the dose–response relationship between the TyG index and uncontrolled hypertension. A positive linear relationship was observed among overall participants (P for non-linearity = 0.526). However, no significant positive association was observed between traditional IR indicator HOMA-IR and OR of uncontrolled hypertension.

**Table 2:** Odds ratios of uncontrolled hypertension by TyG index in the NHANES 1999–2018

Model	Hypertension Uncontrolled, No/Total No	Odds ratios (95% CI)				
		Quartiles of TyG index				
		Quartile 1	Quartile 2	Quartile 3	Quartile 4	P for trend
Overall	3890/8513					
Unadjusted		1 [Reference]	1.07 (0.91, 1.26)	1.14 (0.97, 1.34)	1.20 (1.02, 1.41)^*^	0.013
Model 1		1 [Reference]	1.07 (0.90, 1.26)	1.13 (0.98, 1.35)	1.20 (1.05, 1.45)^*^	0.020
Model 2		1 [Reference]	1.14 (0.94, 1.35)	1.28 (1.06, 1.52)^*^	1.49 (1.21, 1.89)^*^	< 0.001
With medication	704/1973					
Unadjusted		1 [Reference]	1.34 (0.93, 1.92)	1.44 (1.02, 2.03)	1.65 (1.17, 2.32)	0.008
Model 1		1 [Reference]	1.46 (1.01, 2.14)^*^	1.55 (1.08, 2.20)^*^	1.92 (1.33, 2.72)^*^	0.003
Model 2		1 [Reference]	1.55 (1.05, 2.24)^*^	1.65 (1.12, 2.38)^*^	2.03 (1.37, 3.11)^*^	0.002
Without medication	3186/6540					
Unadjusted		1 [Reference]	1.06 (0.88, 1.28)	1.14 (0.95, 1.38)	1.18 (0.98, 1.42)	0.031
Model 1		1 [Reference]	1.03 (0.88, 1.25)	1.13 (0.93, 1.34)	1.16 (0.96, 1.42)	0.066
Model 2		1 [Reference]	1.04 (0.86, 1.26)	1.19 (0.96, 1.46)	1.38 (1.10, 1.72)^*^	0.002

Model 1: Adjusted for age (continuous), sex (male or female), race (Mexican American, non-Hispanic Black, non-Hispanic White, Other Hispanic, or Other Race), education (less than high school, high school, or more than high school), PIR (continuous)

Model 2: Further adjusted for body mass index (continuous), MET (continuous), smoking status (never, former, or current), alcohol consumption (never, former, mild, moderate, or heavy), diabetes (no or yes), hyperlipidemia (no or yes), cardiovascular disease (no or yes), chronic kidney disease (no or yes), anti-hypertensive agents (no or yes), anti-diabetic agents (no or yes), anti-hyperlipidemia agents (no or yes)

TyG index, triglyceride-glucose index; NHANES, National Health and Nutrition Examination Survey

^*^P-value < 0.05

To further analyze the association between the IR and uncontrolled hypertension, participants were stratified according to whether taking anti-hypertensive medication. The characteristic of two group participants is showed in Additional file 1: Tables S1, S2. The positive association between TyG index and uncontrolled hypertension was stronger in participants taking anti-hypertensive medication. During participants taking anti-hypertensive medication, the OR for participants in the second, third, and highest quartile of TyG index were 1.55 (95% CI 1.05–2.24), 1.65 (95% CI 1.12–2.38), and 2.03 (95% CI 1.37–3.11) in the fully adjusted model, respectively (Table 2).

Similar results presented among the participants without anti-hypertensive medication using, the OR were 1.38 (95% CI 1.10–1.72) for the highest quartile of TyG index compared to the reference group in Model 2 (Table 2). Figure 2 also showed restricted cubic splines indicating the dose–response relationship between TyG index and uncontrolled hypertension with or without taking anti-hypertensive medication. We observed a positive linear relationship among participants taking anti-hypertensive medication (P for non-linearity = 0.186) (Fig. 2b) and participants without anti-hypertensive medication (P for non-linearity = 0.379) (Fig. 2c). In contrast, When the analyses were conducted using HOMA-IR to replace TyG index, no significant positive association was observed (Additional file 1: Table S3).

### Association between TyG index and blood presssure control level during different subgroups

**Fig. 3:**
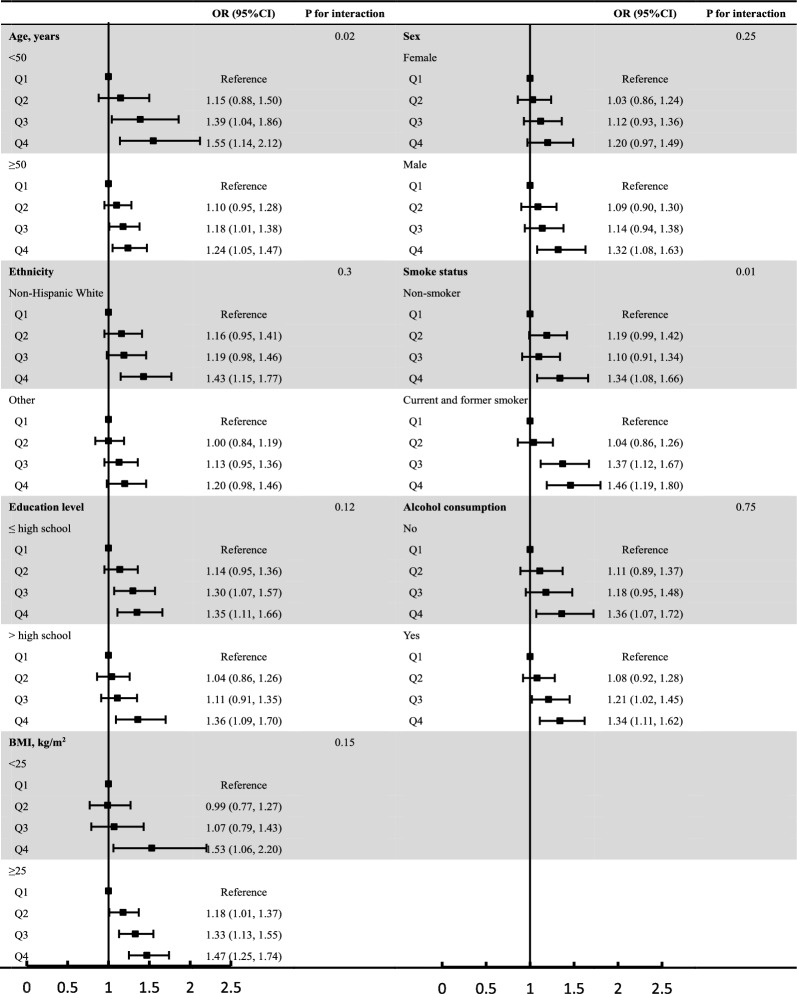
Subgroup analyses for the association of TyG index and uncontrolled hypertension. Adjusted for age, sex, ethnicity, education, BMI, smoking status, alcohol consumption, diabetes, hyperlipidemia, cardiovascular disease, chronic kidney disease, anti-hypertensive agents, anti-diabetic agents, anti-hyperlipidemia agents other than variables for stratification

Furtherly, results of subgroup analyses between TyG index and uncontrolled hypertension stratified by sex, ethnicity, education level, alcohol consumption, BMI, diabetes, and comorbidities (hyperlipidemia, CVD, and CKD) are presented in Fig. 3 and Additional file 1: Fig. S1. Obviously, participants in the highest quartile of TyG index had higher odds of uncontrolled hypertension across various subgroups. There was a significant interaction between age (P for interaction = 0.02) and smoke status (P for interaction = 0.01). No significant interactions between the TyG index and other covariates.

### Association between IR and arterial stiffness

Table 3 shows the regression coefficient (β) of the association between IR and arterial stiffness. The result showed that TyG index was positively associated with ePWV in unadjusted model and full adjusted model 2. After adjusted for all covariates, a 1-unit increase of TyG index was associated with 0.04 m/s increase level of ePWV (P = 0.039). When the analyses were conducted using traditionl IR indicators HOMA-IR and HOMA-IS in place of TyG index, no significant association was observed (Table 3). Meanwhile, this result was consistent with those of the fitting curve (Additional file 1: Fig. S2).

**Table 3:** Beta between ePWV by TyG index in the NHANES 1999–2018

Variables	Beta (95% CI)	P value
TyG index		
Unadjusted	0.08 (0.01, 0.15)	0.028
Model 1	− 0.03 (− 0.07, 0.00)	0.059
Model 2	0.04 (0.00, 0.08)	0.039
HOMA-IR		
Unadjusted	− 0.01 (− 0.02, − 0.01)	< 0.001
Model 1	− 0.01 (− 0.01, − 0.00)	< 0.001
Model 2	− 0.00 (− 0.00, 0.00)	0.103
HOMA-IS		
Unadjusted	0.21 (0.13, 0.29)	< 0.001
Model 1	0.10 (0.07, 0.14)	< 0.001
Model 2	0.03 (− 0.01, 0.07)	0.163

Model 1: Adjusted for age (continuous), sex (male or female), race (Mexican American, non-Hispanic Black, non-Hispanic White, Other Hispanic, or Other Race), education (less than high school, high school, or more than high school), PIR grade (continuous)

Model 2: Further adjusted for body mass index (continuous), MET (continuous), smoking status (never, former, or current), alcohol consumption (never, former, mild, moderate, or heavy), diabetes (no or yes), hyperlipidemia (no or yes), cardiovascular disease (no or yes), chronic kidney disease (no or yes), anti-hypertensive agents (no or yes), anti-diabetic agents (no or yes), anti-hyperlipidemia agents (no or yes)

## Discussion

Our analysis of hypertensive adults enrolled in 1999–2018 NHANES revealed that the TyG index is superior than HOMA-IR in identify uncontrolled hypertension and arterial stiffness. Specifically, when compared to those with a TyG index lower than 8.37, individuals with a TyG index higher than 8.80 were found to have a less favorable rate of blood pressure goal achievement and odds of unsatisfactory blood pressure control that were 28–49% higher among the overall study population. This association was substantially confirmed after adjusted for covariates and even more pronounced in individual taking anti-hypertensive medication. Moreover, TyG index was positively associated with ePWV, whereas HOMA-IR and HOMA-IS showed no significant positive association with ePWV. These results suggest that the TyG index may be an ideal IR indicator for identifying hypertensive patients with high risk of uncontrolled hypertension and elevated arterial stiffness.

IR is linked with dysmetabolic conditions and not only plays a role in the development of diabetes but also contributes to the progression of hypertension and other CVD [37, 38]. Meanwhile, Metabolic syndrome has been been reported to be associated with arterial stiffness [39]. HOMA-IR is a classic used method for assessing IR [40]. However, the use of HOMA-IR is limited in individuals receiving insulin treatment or have beta cells dysfunction [41]. In this regard, the TyG has been developed and shown to be more representative, reliable, and cost-effective than HOMA-IR [20, 41]. In addition, quite a few studies have shown that the TyG index is a risk factor for CVD as well as associated with poor CVD prognosis [19, 25–29, 42–44]. A large cohort study found that early TyG index accumulation increased risk of CVD and all-cause mortality [28]. Furthermore, a meta-analysis showed a potential linear dose–response association was found between the TyG index and CVD incidence [26]. For every unit increase in the TyG index, the risk of coronary heart disease and CVD increases by 35 and 23%, respectively.

The association between the TyG index and hypertension as well as arterial stiffness has been discussed in previous literature. In a South Korean study enrolled 15,721 normotensive adults, Lee et al. [43] found that high TyG index was positively correlated with the risk of increased blood pressure. Meanwhile, Zhu et al. conducted a study among 47,808 participants aged over 40 years, reporting that a high TyG index had 1.33-fold higher odds of hypertension than a low TyG index after lipid and glycemic parameters were well-controlled [19]. In addition, results from a longitudinal cohort indicated that BMI and TyG index have a mutual effect, which is crucial for the development of hypertension [44]. Moreover, A meta-analysis included twenty-six observational studies involving 87,307 participants observed that elevated TyG index is associated with an increased risk of arterial stiffness and CAC. Although these findings provide direct evidence of a relationship between hypertension and the TyG index during whole population, there is limited information available regarding the relationship between the TyG index and uncontrolled hypertension and arterial stiffness.

To the best of our knowledge, this is the first large-scale population study to reveal a significant correlation between the TyG index and uncontrolled hypertension and arterial stiffness. Previous studies have suggested a connection between hyperinsulinemia and blood pressure controlled [45–48]. Two studies showed that IR and poor glycemic control are independently and positively associated with the early morning blood pressure surge which is an independent risk factor for cardiovascular events [45, 47]. The finding of Mioni et al. suggested that the variation of blood pressure is more affected by hyperinsulinemia and/or IR than obesity in patients with polycystic ovary syndrome [46]. Moreover, a retrospective study enrolled 4551 Caucasian adults found that insufficient control of blood pressure is associated with the presence of metabolic syndrome, and patients with metabolic syndrome were 43% more likely to have uncontrolled hypertension than those without [49]. The consistency of these results supports the validity of our findings. In addition, we found that TyG index is associated with ePWV in participants with hypertension, whereas traditional IR indicators, HOMR-IR and HOMR-IS, are not. These results suggested that TyG index is a more sensitive indicator for arterial stiffness among participants with hypertension. In the present study, the TyG index appears to have potential as an indicator of uncontrolled hypertension and arterial stiffness. In real-world clinical practice, the TyG index can be calculated in a cost-effective manner using routine blood biochemical tests.

Although the pathophysiological mechanisms underlying the association between the TyG index and uncontrolled hypertension and arterial stiffness remain unclear, there are several factors that may contribute to this association. First, since the TyG index is the product of fasting triglycerides and fasting glucose, people with a higher TyG index may have more dietary and lifestyle risk factors and less health care awareness. In our study, individuals with higher TyG index were more likely to be smokers, overweight, and have higher prevalence rates of diabetes, hyperlipidemia, cardiovascular disease, and chronic kidney disease. Second, as mentioned earlier, the TyG index is a favorable biomarker of IR which is associated with chronic inflammation, oxidative stress, and endothelial dysfunction of the vascular wall [38, 50]. All of these contribute to the development and progression of hypertension and stiffness [22]. Both aging and smoke are strongly associated with arterial stiffness which may contribute to the result in subgroup analysis [14]. In addition, IR is associated with increased renal sodium reabsorption [51], as well as the inappropriate activation of the sympathetic nervous system and the renin–angiotensin–aldosterone system [51–53]. Thus, a higher TyG index may potentially hinder the effectiveness of certain antihypertensive medications, such as angiotensin-converting enzyme inhibitors, calcium channel blockers, and diuretics. Previous studies have shown that ePWV shares similar predictive value with the measured cf-PWV, and it independently predicts cardiovascular events independently of traditional cardiovascular risk factors, in hypertensive patients, regardless of their antihypertensive treatment status [16, 34]. Nonetheless, ePWV is derived through calculations involving age and MBP, rather than direct measurement. It remains to be substantiated through further research whether the interplay between IR and arterial stiffening is mediated by hypertension and aging.

A major strength of our study is that the results were derived from a large and representative sample of US adults which positively influences the research. However, our study also bears some limitations. First, as a cross-sectional observational study, we only established association, rather than causation, between the TyG index and uncontrolled hypertension as well as arterial stiffness. Second, arterial stiffness was estimated by the use of a standardized calculation and it was not measured using the gold standard reference method in this study. Finally, even though we adjusted for a variety of covariates, the possibility of unknown confounders may not be ruled out.

## Conclusions

In conclusion, elevated levels of the TyG index were were positive associated with prevalence of uncontrolled hypertension and arterial stiffness among US adults with hypertension.

### Supplementary Information

**Additional file 1: Fig. S1.**. Subgroup analyses for the association of TyG index and uncontrolled hypertension stratified by comorbidities. Adjusted for age, sex, ethnicity, education, BMI, smoking status, alcohol consumption, diabetes, hyperlipidemia, cardiovascular disease, chronic kidney disease, anti-hypertensive agents, anti-diabetic agents, anti-hyperlipidemia agents other than variables for stratification. Abbreviations: TyG, triglyceride glucose; Q, quartile; DM, diabetes mellitus; CVD, cardiovascular disease; CKD, chronic kidney disease; OR, odds ratios; CI, confidence interval. **Fig. S2.** Association between TyG index and ePWV. The solid line and dashed line represent the estimated values and their corresponding 95% confdence interval. Adjustment factors included age (continuous), sex (male or female), race (Mexican American, non-Hispanic Black, non-Hispanic White, Other Hispanic, or Other Race), education (less than high school, high school, or more than high school), PIR (continuous), body mass index (continuous), MET (continuous), smoking status (never, former, or current), alcohol consumption (never, former, mild, moderate, or heavy), diabetes (no or yes), hyperlipidemia (no or yes), cardiovascular disease (no or yes), chronic kidney disease (no or yes), anti-hypertensive agents (no or yes), anti-diabetic agents (no or yes), anti-hyperlipidemia agents (no or yes). **Table S1.** Baseline Characteristics of Participants with Hypertension Taking Anti-Hypertensive Agents in the NHANES 1999–2018. **Table S2.** Baseline characteristics of hypertensive participants without anti-hypertensive medication in the NHANES 1999–2018. **Table S3.** Odds ratios of uncontrolled hypertension by HOMA-IR in the NHANES 1999–2018.

## Data Availability

The data used in this study are openly available from the Centers for Disease Control and Prevention at https://www.cdc.gov/nchs/nhanes/index.htm.

## Abbreviations

TyG: Triglyceride glucose
NHANES: National Health and Nutrition Examination Survey
ePWV: Estimated pulse wave velocity
PIR: Family income to poverty ratio
CVD: Cardiovascular disease
CKD: Chronic kidney disease
IR: Insulin resistance
LDL: Low-density lipoprotein
HDL: High-density lipoprotein
BMI: Body mass index
SBP: Systolic blood pressure
DBP: Diastolic blood pressure
HbA1c: Hemoglobin type A1C
eGFR: Estimation of glomerular filtration rate
CKD-EPI: Chronic Kidney Disease Epidemiology Collaboration
RCS: Restricted cubic spline
Q: Quartiles
OR: Odds ratios
CI: Confidential intervals
