# Supplementary material for: Association between insulin resistance and uncontrolled hypertension and arterial stiffness among US adults: a population-based study
Source: Cardiovasc Diabetol. 2023 Nov 9;22:311. doi: 10.1186/s12933-023-02038-5 (PMC10637002; doi:10.1186/s12933-023-02038-5)
Supplement: Supplementary file 1 — Additional file 1: Fig. S1.. Subgroup analyses for the association of TyG index and uncontrolled hypertension stratified by comorbidities. Adjusted for age, sex, ethnicity, education, BMI, smoking status, alcohol consumption, diabetes, hyperlipidemia, cardiovascular disease, chronic kidney disease, anti-hypertensive agents, anti-diabetic agents, anti-hyperlipidemia agents other than variables for stratification. Abbreviations: TyG, triglyceride glucose; Q, quartile; DM, diabetes mellitus; CVD, cardiovascular disease; CKD, chronic kidney disease; OR, odds ratios; CI, confidence interval. Fig. S2. Association between TyG index and ePWV. The solid line and dashed line represent the estimated values and their corresponding 95% confdence interval. Adjustment factors included age (continuous), sex (male or female), race (Mexican American, non-Hispanic Black, non-Hispanic White, Other Hispanic, or Other Race), education (less than high school, high school, or more than high school), PIR (continuous), body mass index (continuous), MET (continuous), smoking status (never, former, or current), alcohol consumption (never, former, mild, moderate, or heavy), diabetes (no or yes), hyperlipidemia (no or yes), cardiovascular disease (no or yes), chronic kidney disease (no or yes), anti-hypertensive agents (no or yes), anti-diabetic agents (no or yes), anti-hyperlipidemia agents (no or yes). Table S1. Baseline Characteristics of Participants with Hypertension Taking Anti-Hypertensive Agents in the NHANES 1999–2018. Table S2. Baseline characteristics of hypertensive participants without anti-hypertensive medication in the NHANES 1999–2018. Table S3. Odds ratios of uncontrolled hypertension by HOMA-IR in the NHANES 1999–2018. [file 12933_2023_2038_MOESM1_ESM.docx]

**Additional files**


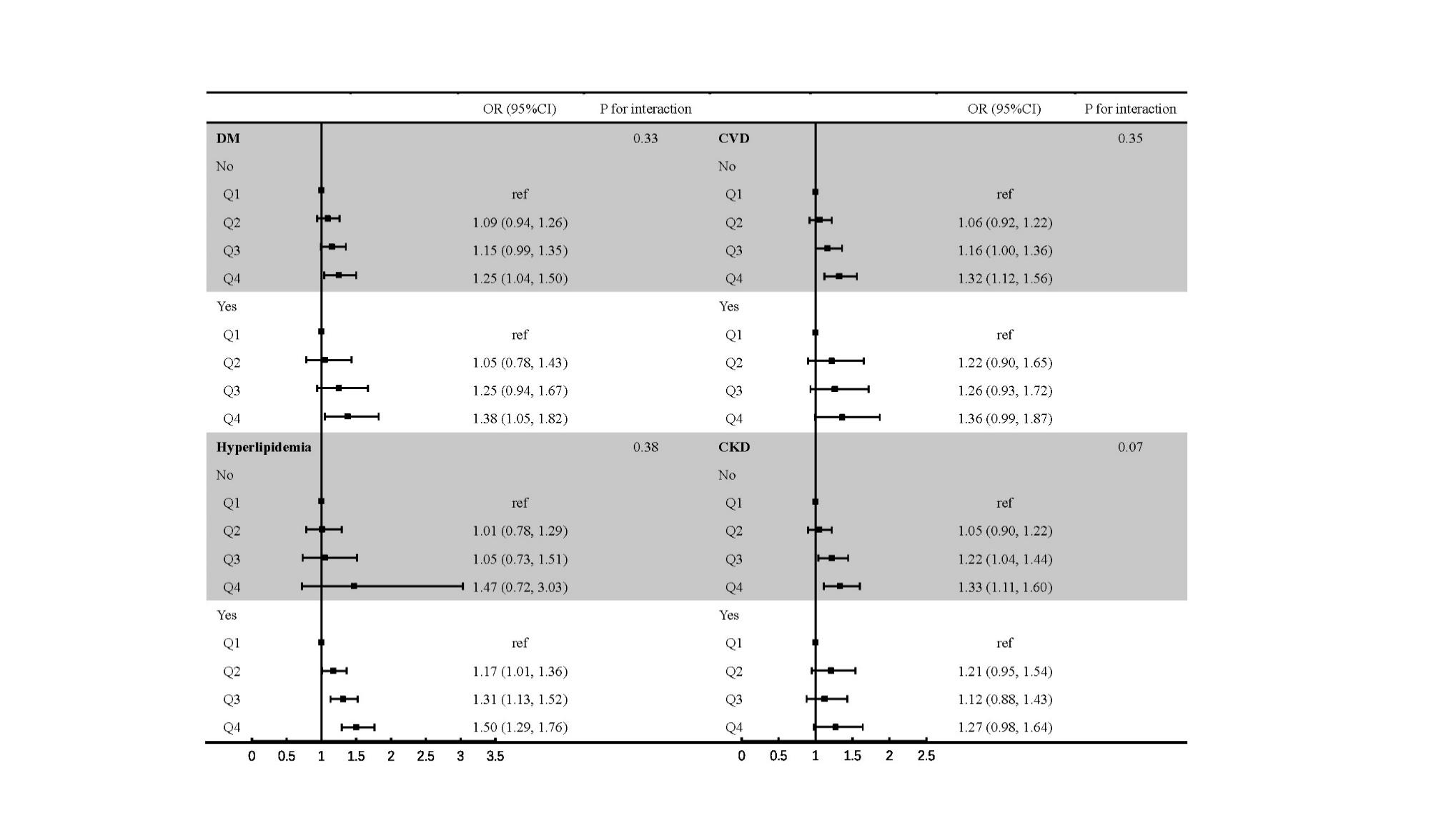


**Fig. S1.** **Subgroup analyses for the association of TyG index and uncontrolled hypertension stratified by comorbidities.**

Adjusted for age, sex, ethnicity, education, BMI, smoking status, alcohol consumption, diabetes, hyperlipidemia, cardiovascular disease, chronic kidney disease, anti-hypertensive agents, anti-diabetic agents, anti-hyperlipidemia agents other than variables for stratification. Abbreviations: TyG, triglyceride glucose; Q, quartile; DM, diabetes mellitus; CVD, cardiovascular disease; CKD, chronic kidney disease; OR, odds ratios; CI, confidence interval.


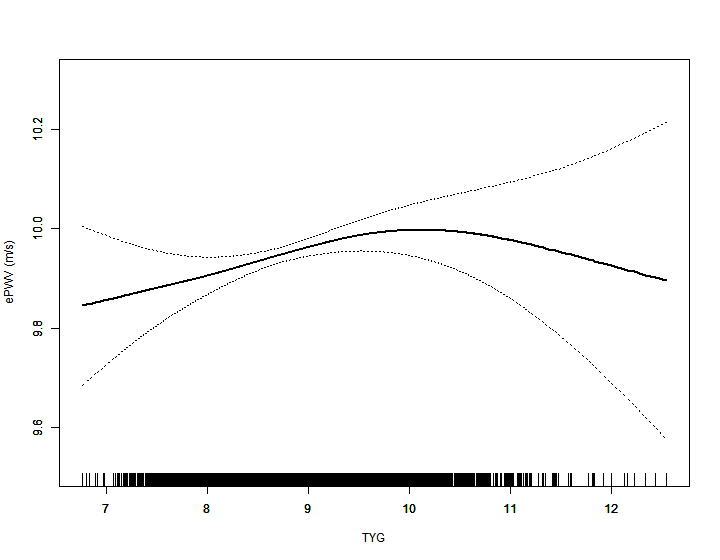


**Fig. S2. Association between TyG index and ePWV.**

The solid line and dashed line represent the estimated values and their corresponding 95% confdence interval. Adjustment factors included age (continuous), sex (male or female), race (Mexican American, non-Hispanic Black, non-Hispanic White, Other Hispanic, or Other Race), education (less than high school, high school, or more than high school), PIR (continuous), body mass index (continuous), MET (continuous), smoking status (never, former, or current), alcohol consumption (never, former, mild, moderate, or heavy), diabetes (no or yes), hyperlipidemia (no or yes), cardiovascular disease (no or yes), chronic kidney disease (no or yes), anti-hypertensive agents (no or yes), anti-diabetic agents (no or yes), anti-hyperlipidemia agents (no or yes).

**Table S1.** Baseline Characteristics of Participants with Hypertension Taking Anti-Hypertensive Agents in the NHANES 1999-2018.

| Characteristics | No. (weighted, %) | | | |
| --- | --- | --- | --- | --- |
|  | Controlled hypertension | Uncontrolled hypertension | Total | P-value |
| TyG index | 8.87 ± 0.03 | 8.99 ± 0.03 | 8.91 ± 0.02 | 0.005 |
| Age, years | 59.08 ± 0.45 | 63.91 ± 0.55 | 60.57 ± 0.37 | < 0.001 |
| Sex |  |  |  | 0.32 |
| Female | 687 (55.78) | 399 (59.02) | 1086 |  |
| Male | 582 (44.22) | 305 (40.98) | 887 |  |
| Ethnicity |  |  |  | 0.01 |
| Non-Hispanic White | 697 (79.15) | 339 (71.53) | 1036 |  |
| Non-Hispanic Black | 310 (11.84) | 202 (16.00) | 512 |  |
| Mexican American | 140 (2.82) | 105 (3.62) | 245 |  |
| Other Race | 56 (3.44) | 26 (5.06) | 82 |  |
| Other Hispanic | 66 (2.74) | 32 (3.79) | 98 |  |
| Education level |  |  |  | 0.01 |
| less than high school | 163 (6.91) | 131 (10.75) | 294 |  |
| high school | 504 (38.96) | 296 (41.37) | 800 |  |
| more than high school | 602 (54.12) | 277 (47.88) | 879 |  |
| Alcohol consumption |  |  |  | 0.002 |
| Never | 208 (13.11) | 142 (19.22) | 350 |  |
| Former | 308 (20.76) | 198 (25.11) | 506 |  |
| Mild | 468 (39.91) | 238 (36.70) | 706 |  |
| Moderate | 157 (16.18) | 61 (10.11) | 218 |  |
| Heavy | 128 (10.04) | 65 (8.86) | 193 |  |
| Smoking status |  |  |  | 0.34 |
| Never | 596 (46.32) | 362 (51.15) | 958 |  |
| Former | 482 (38.44) | 245 (35.74) | 727 |  |
| Current | 191 (15.24) | 97 (13.11) | 288 |  |
| BMI, mean, kg/m^2^ | 31.40 ± 0.28 | 31.0 2± 0.35 | 31.28 ± 0.23 | 0.37 |
| SBP, mm/Hg | 120.88 ± 0.39 | 152.62 ± 0.77 | 130.68 ± 0.53 | < 0.001 |
| DBP, mm/Hg | 68.38 ± 0.46 | 75.51 ± 0.84 | 70.58 ± 0.45 | < 0.001 |
| Blood glucose | 113.06 ± 1.02 | 117.1 1± 1.79 | 114.31 ± 0.85 | 0.06 |
| Insulin, uu/mL | 18.12 ± 0.94 | 16.53 ± 0.69 | 17.63 ± 0.65 | 0.2 |
| HbA1c, % | 5.86 ± 0.03 | 5.93 ± 0.04 | 5.88 ± 0.03 | 0.22 |
| Triglyceride, mg/dL | 152.94 ± 4.28 | 167.60 ± 6.12 | 157.46 ± 3.47 | 0.05 |
| Cholesterol, mg/dL | 197.78 ± 1.73 | 204.61 ± 1.99 | 199.89 ± 1.46 | 0.004 |
| High density lipoprotein, mg/dl | 52.47±0.59 | 53.20±0.70 | 52.70±0.47 | 0.41 |
| Hypertension resistance |  |  |  | < 0.001 |
| No | 1269 (100.00) | 654 (94.12) | 1923 |  |
| Yes | 0 (0.00) | 50 (5.88) | 50 |  |
| Anti-diabetic agents |  |  |  | 0.31 |
| No | 991 (82.69) | 535 (80.25) | 1526 |  |
| Yes | 278 (17.31) | 169 (19.75) | 447 |  |
| Anti-hyperlipidemia agents |  |  |  | 0.12 |
| No | 755 (60.44) | 466 (64.55) | 5778 |  |
| Yes | 514 (39.56) | 238 (35.45) | 2735 |  |
| Diabetes |  |  |  | 0.46 |
| No | 820 (70.94) | 450 (68.91) | 1270 |  |
| Yes | 449 (29.06) | 254 (31.09) | 703 |  |
| Hyperlipidemia |  |  |  | 0.51 |
| No | 187 (13.79) | 105 (12.48) | 292 |  |
| Yes | 1082 (86.21) | 599 (87.52) | 1681 |  |
| CVD |  |  |  | 0.58 |
| No | 982 (79.58) | 533 (78.40) | 6798 |  |
| Yes | 287 (20.42) | 171 (21.60) | 1715 |  |
| CKD |  |  |  | < 0.001 |
| No | 878 (74.41) | 396 (61.92) | 1274 |  |
| Yes | 391 (25.59) | 308 (38.08) | 699 |  |
| All-cause mortality |  |  |  | < 0.001 |
| Alive | 878 (73.68) | 377 (60.20) | 1255 |  |
| Death | 391 (26.32) | 327 (39.80) | 718 |  |
| CVD mortality |  |  |  | < 0.001 |
| Alive | 1140 (91.07) | 574 (84.53) | 1714 |  |
| Death | 129 (8.93) | 130 (15.47) | 259 |  |

All values were presented as mean ± SE, or counts (weighted, proportion).

TyG index, triglyceride-glucose index; NHANES, National Health and Nutrition Examination Survey; BMI, body mass index; SBP, systolic blood pressure; DBP, diastolic blood pressure; HbA1c, hemoglobin type A1C; CVD, cardiovascular disease; CKD, chronic kidney disease.

**Table S2.** Baseline characteristics of hypertensive participants without anti-hypertensive medication in the NHANES 1999-2018.

| Characteristics | No. (weighted, %) | | | |
| --- | --- | --- | --- | --- |
|  | Controlled hypertension | Uncontrolled  hypertension | Total | P-value |
| TyG index | 8.80 ± 0.02 | 8.83 ± 0.02 | 8.82 ± 0.01 | 0.21 |
| Age, years | 54.11 ± 0.36 | 58.04 ± 0.39 | 55.84 ± 0.28 | < 0.001 |
| Sex |  |  |  | 0.02 |
| Female | 1653 (49.16) | 1484 (45.38) | 3137 |  |
| Male | 1701 (50.84) | 1702 (54.62) | 3403 |  |
| Ethnicity |  |  |  | < 0.001 |
| Non-Hispanic White | 1681 (73.85) | 1372 (68.66) | 3053 |  |
| Non-Hispanic Black | 685 (9.77) | 779 (13.94) | 1464 |  |
| Mexican American | 446 (5.33) | 544 (6.80) | 990 |  |
| Other Race | 263 (6.77) | 235 (5.86) | 498 |  |
| Other Hispanic | 279 (4.29) | 256 (4.74) | 535 |  |
| Education level |  |  |  | < 0.001 |
| less than high school | 382 (5.68) | 527 (8.50) | 909 |  |
| high school | 1313 (36.44) | 1339 (41.42) | 2652 |  |
| more than high school | 1659 (57.88) | 1320 (50.07) | 2979 |  |
| Alcohol consumption |  |  |  | 0.01 |
| Never | 455 (10.02) | 518 (13.75) | 973 |  |
| Former | 752 (18.43) | 696 (18.63) | 1448 |  |
| Mild | 1171 (39.86) | 1101 (37.13) | 2272 |  |
| Moderate | 422 (14.79) | 364 (13.18) | 786 |  |
| Heavy | 554 (16.90) | 507 (17.32) | 1061 |  |
| Smoking status |  |  |  | < 0.001 |
| Never | 1623 (47.76) | 1640 (50.54) | 3263 |  |
| Former | 1018 (29.85) | 988 (32.61) | 2006 |  |
| Current | 713 (22.40) | 558 (16.85) | 1271 |  |
| BMI, kg/m^2^ | 30.87 ± 0.14 | 30.12 ± 0.17 | 30.54 ± 0.12 | < 0.001 |
| SBP, mm/Hg | 120.91 ± 0.25 | 149.99 ± 0.34 | 133.71 ± 0.32 | < 0.001 |
| DBP, mm/Hg | 69.72 ± 0.27 | 79.63 ± 0.41 | 74.09 ± 0.27 | < 0.001 |
| Blood glucose | 113.02 ± 0.87 | 113.07 ± 0.98 | 113.04 ± 0.70 | 0.96 |
| Insulin, uu/mL | 15.03 ± 0.34 | 14.35 ± 0.34 | 14.73 ± 0.24 | 0.43 |
| HbA1c, % | 5.83 ± 0.03 | 5.81 ± 0.03 | 5.82 ± 0.02 | 0.22 |
| Triglyceride, mg/dL | 146.50 ± 2.61 | 149.62 ± 2.37 | 147.87 ± 1.97 | 0.32 |
| Cholesterol, mg/dL | 192.69 ± 1.03 | 204.54 ± 1.07 | 197.91 ± 0.76 | < 0.001 |
| High density lipoprotein, mg/dl | 51.48 ± 0.39 | 54.81 ± 0.48 | 52.95 ± 0.31 | < 0.001 |
| Anti-diabetic agents |  |  |  | < 0.001 |
| No | 2689 (83.84) | 2709 (88.45) | 5398 |  |
| Yes | 1220 (34.78) | 763 (23.98) | 1142 |  |
| Anti-hyperlipidemia agents |  |  |  | < 0.001 |
| No | 2134 (65.22) | 2423 (76.02) | 4557 |  |
| Yes | 514 (39.56) | 238 (35.45) | 1983 |  |
| Diabetes |  |  |  | 0.12 |
| No | 2300 (74.29) | 2265 (76.29) | 4565 |  |
| Yes | 1054 (25.71) | 921 (23.71) | 1975 |  |
| Hyperlipidemia |  |  |  | 0.89 |
| No | 603 (17.74) | 568 (17.57) | 1171 |  |
| Yes | 2751 (82.26) | 2618 (82.43) | 5369 |  |
| CVD |  |  |  | 0.002 |
| No | 2633 (82.06) | 2650 (85.29) | 5283 |  |
| Yes | 721 (17.94) | 536 (14.71) | 1257 |  |
| CKD |  |  |  | < 0.001 |
| No | 2508 (80.06) | 2116 (72.19) | 4624 |  |
| Yes | 846 (19.94) | 1070 (27.81) | 1916 |  |
| All-cause mortality |  |  |  | < 0.001 |
| Alive | 2709 (85.52) | 2309 (77.73) | 5018 |  |
| Death | 645 (14.48) | 877 (22.27) | 1522 |  |
| Cardiovascular mortality |  |  |  | < 0.001 |
| Alive | 3154 (95.83) | 2875 (92.40) | 6029 |  |
| Death | 200 (4.17) | 311 (7.60) | 511 |  |

All values were presented as mean ± SE, or counts (weighted, proportion).

TyG index, triglyceride-glucose index; NHANES, National Health and Nutrition Examination Survey; BMI, body mass index; SBP, systolic blood pressure; DBP, diastolic blood pressure; HbA1c, hemoglobin type A1C; CVD, cardiovascular disease; CKD, chronic kidney disease.

**Table S3.** Odds ratios of uncontrolled hypertension by HOMA-IR in the NHANES 1999-2018

|  | Hypertension Uncontrolled, No/Total No. | Odds ratios (95% CI) | | | | |
| --- | --- | --- | --- | --- | --- | --- |
|  |  | Quartiles of HOMA-IR | | | | |
| Model |  | Quartile 1  0.35-1.80 | Quartile 2  1.80-3.05 | Quartile 3  3.05-5.30 | Quartile 4  5.30-269.41 | P for trend |
| Overall | 3890/8513 |  |  |  |  |  |
| Unadjusted |  | 1 [Reference] | 0.95 (0.85, 1.08) | 0.74 (0.65, 0.83) ^*^ | 0.73 (0.65, 0.83) ^*^ | < 0.001 |
| Model 1 |  | 1 [Reference] | 0.94 (0.83, 1.07) | 0.72 (0.64, 0.82) ^*^ | 0.71 (0.63, 0.81) ^*^ | < 0.001 |
| Model 2 |  | 1 [Reference] | 1.00 (0.88, 1.14) | 0.81 (0.70, 0.92) ^*^ | 0.87 (0.74, 1.01) | 0.054 |
| With medication | 704/1973 |  |  |  |  |  |
| Unadjusted |  | 1 [Reference] | 1.19 (0.90, 1.57) | 0.70 (0.53, 0.93) | 0.97 (0.74, 1.28) | 0.507 |
| Model 1 |  | 1 [Reference] | 1.20 (0.90, 1.59) | 0.69 (0.52, 0.92) ^*^ | 1.00 (0.76, 1.33) | 0.692 |
| Model 2 |  | 1 [Reference] | 1.25 (0.93, 1.68) | 0.72 (0.53, 0.98) ^*^ | 1.06 (0.75, 1.49) | 0.957 |
| Without medication | 3186/6540 |  |  |  |  |  |
| Unadjusted |  | 1 [Reference] | 0.93 (0.81, 1.07) | 0.79 (0.69, 0.91) ^*^ | 0.72 (0.62, 0.82) ^*^ | < 0.001 |
| Model 1 |  | 1 [Reference] | 0.93 (0.81, 1.06) | 0.80 (0.69, 0.92) ^*^ | 0.70 (0.61, 0.81) ^*^ | < 0.001 |
| Model 2 |  | 1 [Reference] | 0.95 (0.82, 1.09) | 0.84 (0.72, 0.98) ^*^ | 0.97 (0.95, 1.00) ^*^ | 0.025 |

^*^ P-value < 0.05

Abbreviations: TyG index, triglyceride-glucose index; NHANES, National Health and Nutrition Examination Survey.

Model 1: Adjusted for age (continuous), sex (male or female), race (Mexican American, non-Hispanic Black, non-Hispanic White, Other Hispanic, or Other Race), education (less than high school, high school, or more than high school), PIR (continuous).

Model 2: Further adjusted for body mass index (continuous), MET (continuous), smoking status (never, former, or current), alcohol consumption (never, former, mild, moderate, or heavy), diabetes (no or yes), hyperlipidemia (no or yes), cardiovascular disease (no or yes), chronic kidney disease (no or yes), anti-hypertensive agents (no or yes), anti-diabetic agents (no or yes), anti-hyperlipidemia agents (no or yes).
